# Associations between polymorphisms in *IL*-*10* gene and the risk of viral hepatitis: a meta-analysis

**DOI:** 10.1186/s13099-020-00372-7

**Published:** 2020-07-28

**Authors:** Yuanyuan Zhang, Lisha Chen, Huixin Chen

**Affiliations:** Department of Digestive Diseases, Huizhou Municipal Center Hospital, No. 41 of North Yuling Road, Huizhou, 516001 China

**Keywords:** Viral hepatitis, Hepatitis B virus (HBV), Hepatitis C virus (HCV), Gene polymorphisms, Meta-analysis

## Abstract

**Background:**

The relationships between polymorphisms in interleukin-10 (*IL*-*10*) gene and the risk of viral hepatitis remain inconclusive. Therefore, the authors conducted so far the very first meta-analysis to robustly assess the relationships between polymorphisms in *IL*-*10* gene and the risk of viral hepatitis by integrating the results of previous works.

**Methods:**

Medline, Embase, Wanfang, VIP and CNKI were searched throughly for eligible studies, and 76 genetic association studies were finally included in this meta-analysis.

**Results:**

We noticed that rs1800871 (− 819 C/T), rs1800872 (− 592 C/A) and rs1800896 (− 1082 G/A) polymorphisms were all significantly associated with the risk of viral hepatitis in Asians, whereas only rs1800896 (− 1082 G/A) polymorphism was significantly associated with the risk of viral hepatitis in Caucasians. In further analyses by disease subtypes, we noticed that the three investigated polymorphisms were all significantly associated with the risk of both HBV and HCV.

**Conclusions:**

This meta-analysis demonstrates that rs1800871 (− 819 C/T), rs1800872 (− 592 C/A) and rs1800896 (− 1082 G/A) polymorphisms may influence the risk of viral hepatitis in Asians, while only rs1800896 (− 1082 G/A) polymorphism may influence the risk of viral hepatitis in Caucasians. In further analyses by disease subtypes, we noticed that the three investigated polymorphisms may influence the risk of both HBV and HCV.

## Background

Viral hepatitis refers to a type of infectious disorder that is caused by hepatitis viruses which include HAV, HBV, HCV, HDV and HEV [1, 2]. In addition to acute liver injury, these hepatitis viruses may also lead to life-threatening conditions such as liver cirrhosis or hepatocellular carcinoma (HCC) [3, 4]. The clinical course of viral hepatitis is resulted from a complex interaction between pathogen, host and environmental factors, some patients may be asymptomatic the whole life, but some patients may eventually develop liver cirrhosis or even HCC [5, 6]. Therefore, there is no doubt that individual anti-viral immunity is vital for the onset and development of viral hepatitis.

Interleukin-10 (IL-10) serves as one of the most important anti-inflammatory and immunosuppressive factor, and it plays a crucial role in regulating anti-viral immune responses [7–9]. Considering the immune-regulatory effects of IL-10, over the last decade, investigators all over the world have repeatedly attempted to explore the relationships between polymorphisms in *IL*-*10* gene and the risk of viral hepatitis, yet the relationships between these polymorphisms and the risk of viral hepatitis are still inconclusive. So a meta-analysis was conducted to robustly analyze the relationships between polymorphisms in *IL*-*10* gene and the risk of viral hepatitis by integrating the results of previous works.

## Methods

The PRISMA guideline was strictly followed by the authors when designing and implementing this study [10].

### Literature search and inclusion criteria

Medline, Embase, Wanfang, VIP and CNKI were throughly searched by the authors with the below terms: (Interleukin-10 OR IL-10 OR Interleukin 10 OR IL 10) AND (Polymorphism OR Polymorphic OR Variation OR Variant OR Mutant OR Mutation OR SNP OR Genotypic OR Genotype OR Allelic OR Allele) AND (Viral hepatitis OR Chronic hepatitis OR Acute hepatitis OR Hepatitis A OR Hepatitis B OR Hepatitis C OR Hepatitis D OR Hepatitis E OR HAV OR HBV OR HCV OR HDV OR HEV). Moreover, we also manually screened the reference lists of retrieved publications to make up for the potential incompleteness of electronic literature searching.

Selection criteria of this meta-analysis were listed below: (1) Studies of case–control or cohort design; (2) Give genotypic or allelic frequencies of *IL*-*10* polymorphisms in cases with viral hepatitis and population-based controls; (3) The full manuscript with required genotypic or allelic frequencies of *IL*-*10* polymorphisms is retrievable or buyable. Articles would be excluded if one of the following three criteria is satisfied: (1) Studies without complete data about genotypic or allelic frequencies of *IL*-*10* polymorphisms in cases with viral hepatitis and population-based controls; (2) Narrative or systematic reviews, meta-analysis or comments; (3) Case series of subjects with viral hepatitis only. If duplicate publications were retrieved from literature search, we would only include the most complete one for integrated analyses.

### Data extraction and quality assessment

The authors extracted the following data items from eligible studies: (1) Last name of the leading author; (2) Publication year; (3) Country and ethnicity of study population; (4) The number of cases with viral hepatitis and population-based controls; (5) Genotypic frequencies of *IL*-*10* polymorphisms in cases with viral hepatitis and population-based controls. Hardy–Weinberg equilibrium was then tested by using genotypic frequencies of *IL*-*10* polymorphisms, and the threshold of derivation from HWE was set at 0.05. The quality of eligible publications was assessed by the Newcastle–Ottawa scale (NOS) [11], and those with scores of 7–9 were considered to be publications of good quality. Two authors extracted data and assessed quality of eligible publications in parallel. A thorough discussion until a consensus is reached would be endorsed in case of any discrepancy between two authors.

### Statistical analyses

All statistical analyses in this meta-analysis were performed by using the Cochrane Review Manager software. Relationships between *IL*-*10* gene polymorphisms and the risk of viral hepatitis were explored by using odds ratio and its 95% confidence interval. The statistically significant p value was set at 0.05. The authors used I^2^ statistics to evaluate heterogeneities among included studies. The authors would use DerSimonian–Laird method, which is also known as the random effect model, to integrate the results of eligible studies if I^2^ is larger than 50%. Otherwise, the authors would use Mantel–Haenszel method, which is also known as the fixed effect model, to integrate the results of eligible studies. Meanwhile, the authors also conduct subgroup analyses by ethnic groups and disease subtypes. Stabilities of integrated results were tested by deleting one eligible study each time, and then integrating the results of the rest of eligible studies. Publication biases were evaluated by assessing symmetry of funnel plots.

## Results

### Characteristics of included studies

**Fig. 1:**
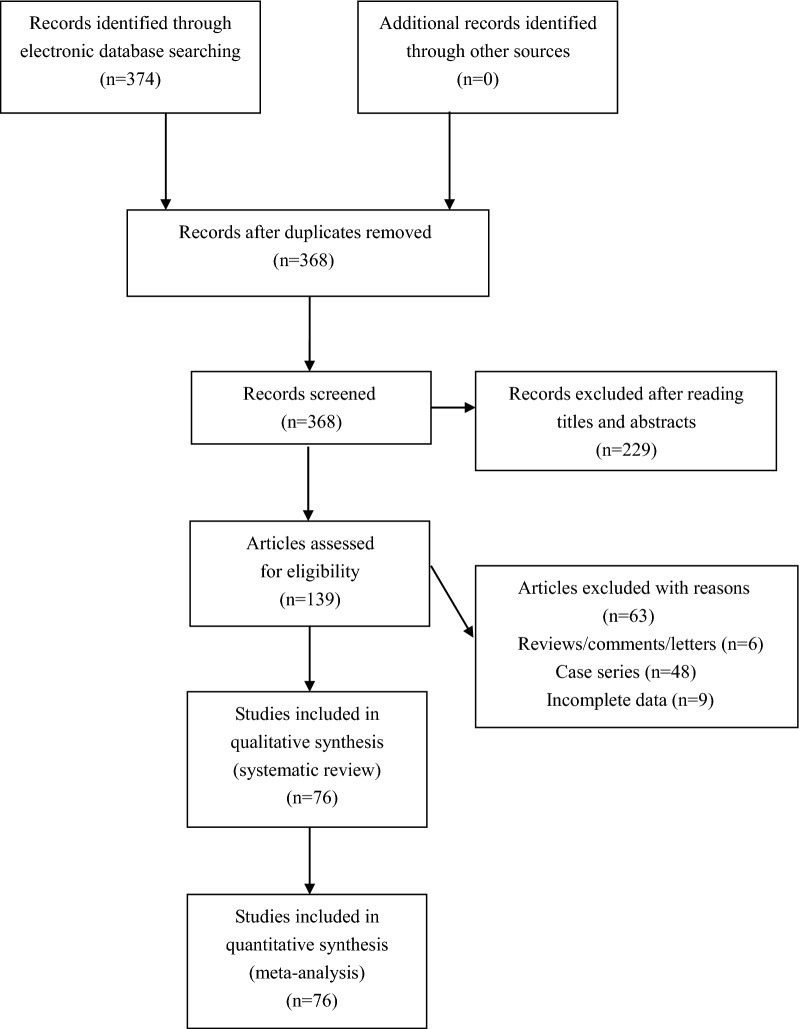
Flowchart of study selection for this meta-analysis. Systematic literature search of the present meta-analysis

Three hundred and seventy-four literatures were retrieved by the authors by using our searching strategy. One hundred and thirty-nine literatures were then selected to screen for eligibility after omitting unrelated and repeated items. Six reviews and 48 case series were further excluded, and another nine literatures without all necessary genotypic or allelic data were further excluded by the authors. Totally 76 studies met the inclusion criteria, and were finally enrolled for integrated analyses (Fig. 1). Data extracted from eligible studies were summarized in Table 1 (Additional file 1).

**Table 1:** The characteristics of included studies

First author, year	Country	Ethnicity	Type of disease	Sample size Case/control	Genotypes (wtwt/wtmt/mtmt)		*p*-value for HWE	NOS score
					Cases	Controls		
rs1800871 − 819 C/T								
Abbas 2009	Egypt	Mixed	HCV	99/62	44/43/12	30/27/5	0.752	7
Afzal 2011	Pakistan	Mixed	HCV	89/99	16/66/7	15/81/3	< 0.001	7
Barrett 2003	Ireland	Caucasian	HCV	92/66	49/38/5	40/22/2	0.621	7
Basturk 2008	Turkey	Caucasian	HBV	50/60	33/15/2	29/22/9	0.175	7
Chen 2007	China	Asian	HCV	72/180	36/32/4	94/73/13	0.819	7
Cheong 2006	Taiwan	Asian	HBV	261/72	133/110/18	35/30/7	0.877	7
Chuang 2009	Taiwan	Asian	HCV	97/46	47/38/12	25/19/2	0.491	7
Constantini 2002	UK	Caucasian	HCV	546/354	NA	NA	NA	7
Cunha 2018	Brazil	Mixed	HCV	132/98	59/54/19	46/41/11	0.685	7
Khan 2014	India	Mixed	HCV	150/150	48/79/23	57/75/18	0.375	8
Komatsu 2014	Japan	Asian	HBV	52/57	24/18/10	25/22/10	0.198	7
Kusumoto 2006	Japan	Asian	HCV	346/114	156/160/30	59/46/9	0.994	7
Li 2006	China	Asian	HBV	122/63	55/52/15	34/21/8	0.118	8
Li 2015	China	Asian	HCV	379/364	176/167/36	178/158/28	0.383	8
Maurya 2018	India	Mixed	Viral hepatitis	80/60	45/29/6	48/10/2	0.138	7
Miyazoe 2002	Japan	Asian	HBV	213/52	153/56/4	38/13/1	0.927	7
Moudi 2016	Iran	Mixed	HBV	221/200	40/163/18	30/162/8	<0.001	8
Peng 2016	China	Asian	HBV	173/181	74/77/22	86/78/17	0.910	8
Pereira 2008	Brazil	Mixed	HCV	128/94	50/60/18	36/48/10	0.305	8
Persico 2006	Italy	Caucasian	HCV	120/110	60/54/6	53/51/6	0.159	8
Qiu 2011	China	Asian	HBV	381/359	170/158/53	181/143/35	0.389	7
Ribeiro 2007	Brazil	Mixed	HBV	30/41	17/12/1	20/16/3	0.935	7
Sepahi 2014	Iran	Mixed	HCV	66/61	32/29/5	20/35/6	0.099	7
Sodsai 2013	Thailand	Asian	HBV	131/142	47/74/10	67/59/16	0.584	8
Sofian 2013	Iran	Mixed	HBV	64/31	26/27/11	16/11/4	0.358	7
Srivastava 2014	India	Mixed	HBV	232/76	111/75/46	29/38/9	0.517	7
Talaat 2014	Egypt	Mixed	HBV	115/119	69/40/6	62/52/5	0.143	7
Tang 2012	China	Asian	HCV	607/885	259/286/62	407/382/96	0.653	8
Tang 2015	China	Asian	HBV	207/56	114/59/34	25/17/14	0.006	7
Vidigal 2002	USA	Mixed	HCV	78/36	53/16/9	20/13/3	0.672	7
Wang 2012	China	Asian	HBV	123/525	40/66/17	205/251/69	0.567	7
Xie 2008	China	Asian	HBV	186/151	78/93/15	73/68/10	0.266	7
Yan 2009	China	Asian	HBV	712/414	334/291/87	231/150/33	0.219	8
Yee 2001	USA	Mixed	HCV	49/50	24/19/6	36/14/0	0.250	7
Zein 2004	USA	Mixed	HCV	58/80	36/17/5	49/25/6	0.279	7
Zhang 2006	China	Asian	HBV	231/135	103/103/25	56/67/12	0.199	8
Zhu 2015	China	Asian	HCV	143/36	56/66/21	18/14/4	0.616	7
rs1800872 − 592 C/A								
Abbas 2009	Egypt	Mixed	HCV	99/62	44/43/12	30/27/5	0.752	7
Afzal 2011	Pakistan	Mixed	HCV	89/99	16/66/7	15/81/3	<0.001	7
Ahmadabadi 2012	Iran	Mixed	HBV	57/100	31/24/2	42/55/3	0.003	8
Barkhash 2017	Russia	Caucasian	HCV	143/203	80/52/11	121/78/4	0.032	7
Barrett 2003	Ireland	Caucasian	HCV	92/66	49/38/5	40/22/2	0.621	7
Basturk 2008	Turkey	Caucasian	HBV	50/60	33/15/2	29/22/9	0.175	7
Cao 2016	China	Asian	HBV	241/254	88/104/49	100/112/42	0.267	7
Chen 2007	China	Asian	HCV	72/180	36/32/4	93/74/13	0.741	7
Chen 2010	China	Asian	HBV	304/361	150/124/30	173/145/43	0.144	7
Cheong 2006	Taiwan	Asian	HBV	261/72	133/110/18	35/30/7	0.877	7
Chuang 2009	Taiwan	Asian	HCV	143/134	73/56/14	65/59/10	0.495	7
Constantini 2002	UK	Caucasian	HCV	546/354	NA	NA	NA	7
Falleti 2007	Italy	Caucasian	HCV	50/96	29/17/4	61/31/4	0.980	7
Gao 2009	China	Asian	HBV	69/74	31/29/9	34/31/9	0.641	7
Gao 2009	China	Asian	HCV	55/74	29/20/6	34/31/9	0.641	7
Gao 2016	China	Asian	HBV	180/85	46/108/26	26/31/18	0.029	8
Jiang 2010	China	Asian	HBV	169/119	75/74/20	51/56/12	0.553	7
Jiang 2013	China	Asian	HBV	250/134	60/130/60	40/62/32	0.409	7
Jiang 2017	China	Asian	HBV	136/289	68/54/14	144/115/30	0.328	8
Karatayli 2014	Turkey	Caucasian	HBV	116/53	63/41/12	29/20/4	0.831	7
Khalil 2017	Egypt	Mixed	HCV	100/120	56/34/10	52/60/8	0.089	7
Komatsu 2014	Japan	Asian	HBV	52/57	23/14/15	26/21/10	0.131	7
Kusumoto 2006	Japan	Asian	HCV	346/114	156/160/30	59/46/9	0.994	7
Li 2003	China	Asian	HBV	95/76	24/58/13	20/43/13	0.218	7
Li 2006	China	Asian	HBV	122/63	55/52/15	34/21/8	0.119	8
Li 2015	China	Asian	HCV	379/364	176/167/36	177/159/28	0.345	8
Mangia 2004	Italy	Caucasian	HCV	270/136	156/90/24	81/55/9	0.003	7
Maurya 2018	India	Mixed	Viral hepatitis	80/60	26/46/8	36/22/2	0.534	7
Miyazoe 2002	Japan	Asian	HBV	213/52	95/91/27	26/20/6	0.483	7
Moudi 2016	Iran	Mixed	HBV	221/200	36/168/17	31/157/12	<0.001	8
Oleksyk 2005	USA	Mixed	HCV	856/398	NA	NA	NA	7
Peng 2006	China	Asian	HBV	340/100	178/130/32	56/36/8	0.519	7
Peng 2016	China	Asian	HBV	173/182	57/81/35	79/81/22	0.860	8
Pereira 2008	Brazil	Mixed	HCV	128/94	50/60/18	36/48/10	0.305	8
Persico 2006	Italy	Caucasian	HCV	120/110	60/54/6	53/51/6	0.159	8
Qiu 2011	China	Asian	HBV	721/359	354/282/85	181/143/35	0.389	7
Ramos 2012	Brazil	Mixed	HCV	161/17	58/60/43	8/5/4	0.120	7
Ren 2017	China	Asian	HBV	250/134	60/130/60	40/62/32	0.409	7
Ribeiro 2007	Brazil	Mixed	HBV	30/41	17/12/1	20/16/3	0.935	7
Sepahi 2014	Iran	Mixed	HCV	66/61	32/29/5	20/35/6	0.099	7
Shaker 2012	Egypt	Mixed	HCV	100/80	35/33/32	36/32/12	0.280	7
Sheneef 2017	Egypt	Mixed	HCV	100/50	58/23/19	25/15/10	0.016	7
Silva 2015	Brazil	Mixed	HCV	245/230	106/110/29	103/97/30	0.347	8
Sodsai 2013	Thailand	Asian	HBV	131/142	47/74/10	67/59/16	0.584	8
Sofian 2013	Iran	Mixed	HBV	86/31	31/42/13	16/11/4	0.358	7
Srivastava 2014	India	Mixed	HBV	202/106	71/102/29	32/42/32	0.033	7
Tang 2012	China	Asian	HCV	623/905	273/289/61	429/370/106	0.058	8
Tseng 2006	Taiwan	Asian	HBV	344/184	169/148/27	90/75/19	0.567	7
Vidigal 2002	USA	Mixed	HCV	78/36	53/16/9	23/10/3	0.239	7
Wang 2008	China	Asian	HBV	335/165	132/169/34	80/64/21	0.156	7
Wang 2012	China	Asian	HBV	123/525	43/63/17	206/250/69	0.615	7
Wu 2010	China	Asian	HBV	175/153	82/67/26	54/77/22	0.515	7
Xiang 2014	China	Asian	HBV	160/124	56/70/34	60/48/16	0.203	7
Xie 2008	China	Asian	HBV	186/151	78/93/15	73/68/10	0.266	7
Yan 2009	China	Asian	HBV	712/414	334/291/87	231/150/33	0.219	8
Yee 2001	USA	Mixed	HCV	49/50	24/19/6	36/14/0	0.250	7
Zein 2004	USA	Mixed	HCV	52/80	37/12/3	52/22/6	0.111	7
Zhang 2006	China	Asian	HBV	396/135	189/168/39	56/67/12	0.199	8
Zhu 2015	China	Asian	HCV	179/705	74/80/25	268/348/89	0.142	7
rs1800896 − 1082 G/A								
Abbas 2009	Egypt	Mixed	HCV	99/62	41/41/17	23/30/9	0.877	7
Afzal 2011	Pakistan	Mixed	HCV	89/99	15/67/7	4/92/3	<0.001	7
Barrett 2003	Ireland	Caucasian	HCV	92/66	20/47/25	20/36/10	0.344	7
Basturk 2008	Turkey	Caucasian	HBV	50/60	17/22/11	38/16/6	0.049	7
Bouzgarrou 2009	Tunisia	Mixed	HCV	100/103	38/43/19	42/49/12	0.687	7
Cao 2016	China	Asian	HBV	241/254	88/112/41	116/111/27	0.954	7
Chen 2007	China	Asian	HCV	72/180	70/2/0	176/4/0	0.880	7
Chen 2010	China	Asian	HBV	304/361	264/37/3	319/40/2	0.544	7
Cheong 2006	Taiwan	Asian	HBV	261/204	225/35/1	173/29/2	0.531	7
Chuang 2009	Taiwan	Asian	HCV	143/133	132/11/0	124/9/0	0.686	7
Conde 2013	Brazil	Mixed	HBV	53/97	27/20/6	47/41/9	0.989	7
Constantini 2002	UK	Caucasian	HCV	546/354	NA	NA	NA	7
Cunha 2018	Brazil	Mixed	HCV	132/98	56/54/22	44/38/16	0.124	7
Dogra 2011	India	Mixed	HCV	70/70	38/22/10	42/25/3	0.764	7
Falleti 2007	Italy	Caucasian	HCV	50/96	17/25/8	28/43/25	0.312	7
Gao 2009	China	Asian	HBV	69/74	42/27/0	57/16/1	0.918	7
Gao 2009	China	Asian	HCV	55/74	32/21/2	57/16/1	0.918	7
Gao 2016	China	Asian	HBV	190/81	177/12/1	63/18/0	0.261	8
Gao 2017	China	Asian	HBV + HCV	179/74	109/68/2	57/16/1	0.918	7
Helal 2014	Egypt	Mixed	HCV	50/50	22/19/9	18/24/8	1.000	7
Jiang 2013	China	Asian	HBV	250/134	189/58/3	102/26/6	0.019	7
Karatayli 2014	Turkey	Caucasian	HBV	161/51	48/86/27	24/25/2	0.144	7
Khan 2014	India	Mixed	HCV	150/150	64/67/19	85/55/10	0.785	8
Knapp 2003	UK	Caucasian	HCV	577/94	183/250/144	27/54/13	0.090	7
Kusumoto 2006	Japan	Asian	HCV	346/114	316/30/0	103/11/0	0.588	7
Li 2006	China	Asian	HBV	62/63	48/14/0	52/11/0	0.448	8
Li 2015	China	Asian	HCV	379/364	323/54/2	310/51/3	0.577	8
Lio 2003	Italy	Caucasian	HCV	60/135	27/15/18	34/86/15	<0.0001	7
Liu 2010	China	Asian	HBV	513/187	416/88/9	160/24/3	0.075	7
Mangia 2004	Italy	Caucasian	HCV	270/145	120/110/40	56/66/23	0.631	7
Maurya 2018	India	Mixed	Vral hepatitis	80/60	65/13/2	46/12/2	0.297	7
Minton 2005	UK	Caucasian	HBV	284/54	77/123/84	18/25/11	0.669	7
Miyazoe 2002	Japan	Asian	HBV	213/52	201/10/2	48/4/0	0.773	7
Moudi 2016	Iran	Mixed	HBV	221/200	72/118/31	100/84/16	0.778	8
Oleksyk 2005	USA	Mixed	HCV	856/398	NA	NA	NA	7
Pár 2014	India	Mixed	HCV	672/92	214/333/125	48/32/12	0.087	8
Pasha 2013	Egypt	Mixed	HCV	440/220	396/44/0	193/27/0	0.332	8
Peng 2006	China	Asian	HBV	340/100	314/23/3	95/5/0	0.798	7
Peng 2016	China	Asian	HBV	173/182	83/74/16	96/74/12	0.653	8
Pereira 2008	Brazil	Mixed	HCV	128/94	56/55/17	38/43/13	0.881	8
Persico 2006	Italy	Caucasian	HCV	120/110	43/51/26	36/56/18	0.628	8
Ren 2017	China	Asian	HBV	250/134	189/58/3	102/26/6	0.019	7
Ribeiro 2007	HBV	Mixed	HBV	30/41	12/16/2	16/20/5	0.743	7
Sepahi 2014	Iran	Mixed	HCV	50/50	20/15/15	39/6/5	<0.001	7
Sheneef 2017	Egypt	Mixed	HCV	100/50	26/43/31	10/35/5	0.003	7
Silva 2015	Brazil	Mixed	HCV	245/230	106/110/29	119/83/28	0.029	8
Sodsai 2013	Thailand	Asian	HBV	130/142	116/13/1	125/17/0	0.448	8
Sofian 2013	Iran	Mixed	HBV	66/31	32/27/7	13/15/3	0.655	7
Srivastava 2014	India	Mixed	HBV	232/76	96/73/63	32/43/1	0.002	7
Talaat 2014	Egypt	Mixed	HBV	115/119	32/53/30	43/61/15	0.352	7
Tang 2012	China	Asian	HCV	626/914	552/74/0	791/123/0	0.029	8
Truelove 2008	USA	Mixed	HBV	45/76	15/24/6	38/32/6	0.837	7
Vidigal 2002	USA	Mixed	HCV	78/36	29/22/27	16/14/6	0.346	7
Wu 2010	China	Asian	HBV	175/153	148/27/0	122/30/1	0.561	7
Xie 2008	China	Asian	HBV	186/151	164/22/0	128/22/1	0.959	7
Yan 2009	China	Asian	HBV	732/414	644/68/0	389/25/0	0.526	8
Yao 2015	China	Asian	HBV	318/318	125/141/52	152/135/31	0.898	7
Zein 2004	USA	Mixed	HCV	52/80	17/18/17	28/32/20	0.087	7
Zhang 2006	China	Asian	HBV	396/135	335/61/0	119/16/0	0.464	8
Zhu 2005	China	Asian	HBV	167/123	115/45/7	81/37/5	0.766	7

*HBV* hepatitis B virus infection, *HCV* hepatitis C virus infection, *wt* wild type, *mt* mutant type, *HWE* Hardy–Weinberg equilibrium, *NOS* Newcastle–ottawa scale, *NA* not available

### Integrated analyses for rs1800871 polymorphism and the risk of viral hepatitis

Thirty-seven eligible literatures assess the relationship between rs1800871 polymorphism and the risk of viral hepatitis. The integrated analyses demonstrated that rs1800871 polymorphism was significantly associated with the risk of viral hepatitis in overall population (dominant comparison: OR = 0.89, p = 0.002; recessive comparison: OR = 1.21, p = 0.004; allele comparison: OR = 0.90, p = 0.0004) and Asians (dominant comparison: OR = 0.84, p = 0.0001; over-dominant comparison: OR = 1.14, p = 0.005; allele comparison: OR = 0.88, p = 0.0002), but not in Caucasians. Further analyses by disease subtypes revealed similar positive results for rs1800871 polymorphism in both HBV and HCV subgroups (see Table 2).

**Table 2:** Meta-analyses results of *IL*-*10* gene polymorphisms and viral hepatitis

Variables	Sample size	Dominant comparison			Recessive comparison			Over-dominant comparison			Allele comparison		
		*p* value	OR (95% CI)	I^2^ statistic (%)	*p* value	OR (95% CI)	I^2^ statistic (%)	*p* value	OR (95% CI)	I^2^ statistic (%)	*p* value	OR (95% CI)	I^2^ statistic (%)
rs1800871 − 819 C/T													
Overall	6835/5679	*0.002*	*0.89 (0.82–0.96)*	28	*0.004*	*1.21 (1.06–1.38)*	0	0.17	1.06 (0.98–1.14)	24	*0.0004*	*0.90 (0.85–0.95)*	27
Asian	4436/3832	*0.0001*	*0.84 (0.76–0.92)*	0	0.09	1.14 (0.98–1.33)	0	*0.005*	*1.14 (1.04–1.25)*	0	*0.0002*	*0.88 (0.82–0.94)*	0
Caucasian	808/590	0.63	1.13 (0.67–1.91)	51	0.37	0.71 (0.33–1.51)	39	0.93	1.02 (0.71–1.46)	0	0.48	1.19 (0.73–1.93)	62
HBV	3504/2734	0.05	0.90 (0.80–1.00)	38	*0.03*	*1.21 (1.02–1.45)*	9	0.51	1.04 (0.93–1.16)	36	*0.02*	*0.91 (0.84-0.98)*	37
HCV	3251/2885	0.05	0.89 (0.80–1.00)	0	0.07	1.20 (0.99–1.45)	0	0.36	1.05 (0.94–1.18)	0	*0.03*	*0.91 (0.83–0.99)*	0
rs1800872 − 592 C/A													
Overall	12121/9873	*0.003*	*0.91 (0.86-0.97)*	25	0.06	1.09 (1.00–1.20)	13	0.07	1.06 (0.99–1.12)	30	*0.003*	*0.93 (0.89–0.98)*	34
Asian	7935/6880	*0.0009*	*0.89 (0.83–0.95)*	9	0.20	1.07 (0.96–1.19)	0	*0.007*	*1.10 (1.03–1.18)*	29	*0.004*	*0.93 (0.88–0.98)*	8
Caucasian	1387/1078	0.70	0.96 (0.78–1.18)	0	0.15	1.36 (0.89–2.08)	34	0.39	0.91 (0.74–1.13)	0	0.19	0.89 (0.76–1.06)	41
HBV	6900/4995	*0.008*	*0.90 (0.83–0.97)*	26	0.43	1.05 (0.93–1.18)	19	*0.02*	*1.10 (1.02–1.19)*	31	*0.04*	*0.94 (0.89–1.00)*	39
HCV	5141/4818	0.35	0.96 (0.87–1.05)	1	*0.05*	*1.17 (1.00–1.37)*	1	0.64	0.98 (0.89–1.08)	12	0.08	0.94 (0.87–1.01)	14
rs1800896 − 1082 G/A													
Overall	13133/8862	*0.02*	*0.87 (0.78–0.98)*	57	*<0.0001*	*1.60 (1.41–1.82)*	26	0.56	0.96 (0.85–1.09)	60	*<0.0001*	*0.83 (0.76–0.90)*	55
Asian	6452/4797	*0.02*	*0.88 (0.79–0.98)*	49	0.48	1.12 (0.82–1.53)	0	0.11	1.09 (0.98–1.22)	40	0.19	0.90 (0.77–1.05)	54
Caucasian	2210/1165	0.65	0.92 (0.64–1.32)	69	*0.009*	*1.67 (1.14–2.46)*	54	0.22	0.80 (0.55–1.15)	72	*0.03*	*0.78 (0.62–0.98)*	64
HBV	6227/4067	*0.01*	*0.82 (0.70-0.96)*	51	< *0.0001*	*1.73 (1.42–2.10)*	27	0.61	1.04 (0.89–1.21)	51	*0.002*	*0.81 (0.71–0.93)*	57
HCV	6647/4661	0.52	0.94 (0.79–1.13)	60	< *0.0001*	*1.52 (1.29–1.80)*	33	0.14	0.87 (0.71–1.05)	66	*0.008*	*0.85 (0.75–0.96)*	51

The values in italic represent there is statistically significant differences between cases and controls

*HBV* Hepatitis B virus infection, *HCV* Hepatitis C virus infection, *OR* Odds ratio, *CI* Confidence interval, *NA* Not available

### Integrated analyses for rs1800872 polymorphism and the risk of viral hepatitis

Fifty-eight eligible literatures assessed the relationship between rs1800872 polymorphism and the risk of viral hepatitis. The integrated analyses demonstrated that rs1800872 polymorphism was significantly associated with the risk of viral hepatitis in overall population (dominant comparison: OR = 0.91, p = 0.003; allele comparison: OR = 0.93, p = 0.003) and Asians (dominant comparison: OR = 0.89, p = 0.0009; over-dominant comparison: OR = 1.10, p = 0.007; allele comparison: OR = 0.93, p = 0.004), but not in Caucasians. Further analyses by disease subtypes revealed similar positive results for rs1800871 polymorphism in both HBV and HCV subgroups (see Table 2).

### Integrated analyses for rs1800896 polymorphism and the risk of viral hepatitis

Fifty-nine eligible literatures assessed the relationship between rs1800896 polymorphism and the risk of viral hepatitis. The integrated analyses demonstrated that rs1800896 polymorphism was significantly associated with the risk of viral hepatitis in overall population (dominant comparison: OR = 0.87, p = 0.02; recessive comparison: OR = 1.60, p < 0.0001; allele comparison: OR = 0.83, p < 0.0001), Asians (dominant comparison: OR = 0.88, p = 0.02) and Caucasians (recessive comparison: OR = 1.67, p = 0.009; allele comparison: OR = 0.78, p = 0.03). Further analyses by disease subtypes revealed similar positive results for rs1800871 polymorphism in both HBV and HCV subgroups (see Table 2).

### Sensitivity analyses

The authors examined stabilities of integrated analyses results by deleting studies that violated HWE, and then integrating the results of the rest of studies. The trends of associations were not significantly altered in sensitivity analyses, which indicated that from statistical perspective, our integrated analyses results were reliable and stable.

### Publication biases

The authors examined potential publication biases in this meta-analysis by assessing symmetry of funnel plots. Funnel plots were found to be overall symmetrical, which indicated that our integrated analyses results were not likely to be severely deteriorated by publication biases.

## Discussion

This meta-analysis, for the first time, robustly assessed associations between polymorphisms in *IL*-*10* gene and the risk of viral hepatitis. The integrated analyses results demonstrated that rs1800871 (− 819 C/T), rs1800872 (− 592 C/A) and rs1800896 (− 1082 G/A) polymorphisms were all significantly associated with the risk of viral hepatitis in Asians, whereas only rs1800896 (− 1082 G/A) polymorphism was significantly associated with the risk of viral hepatitis in Caucasians. In further analyses by disease subtypes, we noticed that the three investigated polymorphisms were all significantly associated with the risk of both HBV and HCV.

The following three points should be considered when interpreting our integrated findings. First, based on the findings of previous observational studies, it is believed that the three investigated *IL*-*10* polymorphisms may alter mRNA expression level of *IL*-*10* gene, impact anti-viral immune responses, and then influence the risk of viral hepatitis [12, 13]. Nevertheless, it should be noted that future experimental studies are still required to reveal the exact molecular mechanisms underlying the observed positive findings of this meta-analysis. Second, we wish to study all polymorphic loci of *IL*-*10* gene. However, our comprehensive literature searching did not reveal sufficient eligible literatures to warrant integrated analyses for other polymorphic loci of *IL*-*10* gene, so we only assessed associations with the risk of viral hepatitis for the three most commonly investigated polymorphisms of *IL*-*10* gene in this meta-analysis. Third, although we aimed to investigate all subtypes of viral hepatitis in this meta-analysis, it is worth noting that the majority of eligible studies were about HBV or HCV. So future studies should continue to explore associations between polymorphisms in *IL*-*10* gene and the risk of other subtypes of viral hepatitis.

The three major limitations of our integrated analyses were listed below. Firstly, our integrated analyses results were only derived from unadjusted pooling of previous works. Without access to raw data of eligible studies, we can only estimate associations based on re-calculations of raw genotypic frequencies, but we have to admit that lack of further adjustment for baseline characteristics may certainly impact reliability of our findings [14]. Secondly, environmental factors may also affect relationships between polymorphisms in *IL*-*10* gene and the risk of viral hepatitis. However, most of the authors only paid attention to genetic associations in their publications, so it is impossible for us to explore genetic-environmental interactions in a meta-analysis based on these previous publications [15]. Thirdly, we did not enroll grey literatures for integrated analyses because these literatures are always incomplete and it is impossible for us to extract all required data items from these literatures or assess their quality through the NOS scale. Nevertheless, considering that we did not include grey literatures for integrated analyses, despite that funnel plots were found to be overall symmetrical, it should be acknowledged that publication biases still may affect the robustness of our integrated analyses results [16].

## Conclusion

In conclusion, this meta-analysis demonstrates that rs1800871 (− 819 C/T), rs1800872 (− 592 C/A) and rs1800896 (− 1082 G/A) polymorphisms may influence the risk of viral hepatitis in Asians, while only rs1800896 (− 1082 G/A) polymorphism may influence the risk of viral hepatitis in Caucasians. In further analyses by disease subtypes, we noticed that the three investigated polymorphisms may influence the risk of both HBV and HCV. However, future studies should continue to investigate associations between polymorphisms in *IL*-*10* gene and the risk of other subtypes of viral hepatitis.

## Supplementary information

**Additional file 1.** References of 76 eligible studies that were included in this meta-analysis

## Data Availability

Not applicable.

## Abbreviations

HBV: Hepatitis B virus
HCV: Hepatitis C virus
IL-10: Interleukin-10
HWE: Hardy–Weinberg equilibrium
NOS: Newcastle–Ottawa scale
OR: Odds ratios
CI: Confidence intervals

## References

[1] Lanini S, Pisapia R, Capobianchi MR, Ippolito G (2018). Global epidemiology of viral hepatitis and national needs for complete control. Expert Rev Anti Infect Ther..

[2] Asrani SK, Devarbhavi H, Eaton J, Kamath PS (2019). Burden of liver diseases in the world. J Hepatol.

[3] Jefferies M, Rauff B, Rashid H, Lam T, Rafiq S (2018). Update on global epidemiology of viral hepatitis and preventive strategies. World J Clin Cases..

[4] Pardee M (2019). Diagnosis and management of hepatitis B and C. Nurs Clin North Am.

[5] Chang ML, Liaw YF (2014). Hepatitis B flares in chronic hepatitis B: pathogenesis, natural course, and management. J Hepatol.

[6] Matsuura K, Tanaka Y (2016). Host genetic variants influencing the clinical course of hepatitis C virus infection. J Med Virol.

[7] Saraiva M, O’Garra A (2010). The regulation of IL-10 production by immune cells. Nat Rev Immunol.

[8] Sanjabi S, Zenewicz LA, Kamanaka M, Flavell RA (2009). Anti-inflammatory and pro-inflammatory roles of TGF-beta, IL-10, and IL-22 in immunity and autoimmunity. Curr Opin Pharmacol.

[9] Rojas JM, Avia M, Martín V, Sevilla N (2017). IL-10: a multifunctional cytokine in viral infections. J Immunol Res..

[10] Moher D, Liberati A, Tetzlaff J, Altman DG, PRISMA group (2009). Preferred reporting items for systematic reviews and meta-analyses: the PRISMA statement. Ann Intern Med..

[11] Stang A (2010). Critical evaluation of the Newcastle-Ottawa scale for the assessment of the quality of nonrandomized studies in meta-analyses. Eur J Epidemiol.

[12] Turner DM, Williams DM, Sankaran D, Lazarus M, Sinnott PJ, Hutchinson IV (1997). An investigation of polymorphism in the interleukin-10 gene promoter. Eur J Immunogenet.

[13] de Oliveira JG, Rossi AF, Nizato DM, Cadamuro AC, Jorge YC, Valsechi MC, Venâncio LP, Rahal P, Pavarino ÉC, Goloni-Bertollo EM, Silva AE (2015). Influence of functional polymorphisms in TNF-α, IL-8, and IL-10 cytokine genes on mRNA expression levels and risk of gastric cancer. Tumour Biol.

[14] Loeffler J, Ok M, Morton OC, Mezger M, Einsele H (2010). Genetic polymorphisms in the cytokine and chemokine system: their possible importance in allogeneic stem cell transplantation. Curr Top Microbiol Immunol.

[15] Opdal SH (2004). IL-10 gene polymorphisms in infectious disease and SIDS. FEMS Immunol Med Microbiol.

[16] Sezgin E, An P, Winkler CA (2019). Host genetics of cytomegalovirus pathogenesis. Front Genet..

