# Supplementary material for: Associations between polymorphisms in IL-10 gene and the risk of viral hepatitis: a meta-analysis
Source: Gut Pathog. 2020 Jul 28;12:36. doi: 10.1186/s13099-020-00372-7 (PMC7385948; doi:10.1186/s13099-020-00372-7)
Supplement: Supplementary file 1 — Additional file 1. References of 76 eligible studies that were included in this meta-analysis [file 13099_2020_372_MOESM1_ESM.docx]

**Additional file 1. References of 76 eligible studies that were included in this meta-analysis**

1. Abbas OM, Abdel-Rahman MH, Omar NA, Badran HM, Amir EM. Interleukin-10 promoter polymorphisms in hepatitis C patients with and without Schistosoma mansoni co-infection. Liver Int. 2009;29(9):1422-1430. doi:10.1111/j.1478-3231.2009.02068.x
2. Afzal MS, Tahir S, Salman A, et al. Analysis of interleukin-10 gene polymorphisms and hepatitis C susceptibility in Pakistan. J Infect Dev Ctries. 2011;5(6):473-479. Published 2011 Jul 4. doi:10.3855/jidc.1338
3. Ahmadabadi BN, Hassanshahi G, Arababadi MK, Leanza C, Kennedy D. The IL-10 promoter polymorphism at position -592 is correlated with susceptibility to occult HBV infection. Inflammation. 2012;35(3):818-821. doi:10.1007/s10753-011-9381-x
4. Barkhash AV, Kochneva GV, Chub EV, Romaschenko AG. Single nucleotide polymorphism rs1800872 in the promoter region of the IL10 gene is associated with predisposition to chronic hepatitis C in Russian population. Microbes Infect. 2018;20(3):212-216. doi:10.1016/j.micinf.2017.11.012
5. Barrett S, Collins M, Kenny C, Ryan E, Keane CO, Crowe J. Polymorphisms in tumour necrosis factor-alpha, transforming growth factor-beta, interleukin-10, interleukin-6, interferon-gamma, and outcome of hepatitis C virus infection. J Med Virol. 2003;71(2):212-218. doi:10.1002/jmv.10472
6. Basturk B, Karasu Z, Kilic M, Ulukaya S, Boyacioglu S, Oral B. Association of TNF-alpha -308 polymorphism with the outcome of hepatitis B virus infection in Turkey. Infect Genet Evol. 2008;8(1):20-25. doi:10.1016/j.meegid.2007.09.001
7. Bouzgarrou N, Hassen E, Farhat K, et al. Combined analysis of interferon-gamma and interleukin-10 gene polymorphisms and chronic hepatitis C severity. Hum Immunol. 2009;70(4):230-236. doi:10.1016/j.humimm.2009.01.019
8. Cao LN, Cheng SL, Liu W. IL10 rs1800896 polymorphism is associated with liver cirrhosis and chronic hepatitis B. Genet Mol Res. 2016;15(1):10.4238/gmr.15017256. Published 2016 Feb 22. doi:10.4238/gmr.15017256
9. Chen TY, Hsieh YS, Wu TT, et al. Impact of serum levels and gene polymorphism of cytokines on chronic hepatitis C infection. Transl Res. 2007;150(2):116-121. doi:10.1016/j.trsl.2007.01.007
10. Chen DQ, Zeng Y, Zhou J, et al. Association of candidate susceptible loci with chronic infection with hepatitis B virus in a Chinese population. J Med Virol. 2010;82(3):371-378. doi:10.1002/jmv.21716
11. Cheong JY, Cho SW, Hwang IL, et al. Association between chronic hepatitis B virus infection and interleukin-10, tumor necrosis factor-alpha gene promoter polymorphisms. J Gastroenterol Hepatol. 2006;21(7):1163-1169. doi:10.1111/j.1440-1746.2006.04304.x
12. Chuang JY, Yang SS, Lu YT, et al. IL-10 promoter gene polymorphisms and sustained response to combination therapy in Taiwanese chronic hepatitis C patients. Dig Liver Dis. 2009;41(6):424-430. doi:10.1016/j.dld.2008.09.017
13. Conde SR, Feitosa RN, Freitas FB, et al. Association of cytokine gene polymorphisms and serum concentrations with the outcome of chronic hepatitis B. Cytokine. 2013;61(3):940-944. doi:10.1016/j.cyto.2013.01.004
14. Constantini PK, Wawrzynowicz-Syczewska M, Clare M, et al. Interleukin-1, interleukin-10 and tumour necrosis factor-alpha gene polymorphisms in hepatitis C virus infection: an investigation of the relationships with spontaneous viral clearance and response to alpha-interferon therapy. Liver. 2002;22(5):404-412. doi:10.1034/j.1600-0676.2002.01553.x
15. Cunha LRD, Vieira DA, Giampietro YG, et al. Interleukin-10 promoter gene polymorphisms are associated with the first major depressive episode in chronic hepatitis C patients. Clin Res Hepatol Gastroenterol. 2019;43(4):417-426. doi:10.1016/j.clinre.2018.11.015
16. Dogra G, Chakravarti A, Kar P, Chawla YK. Polymorphism of tumor necrosis factor-α and interleukin-10 gene promoter region in chronic hepatitis C virus patients and their effect on pegylated interferon-α therapy response. Hum Immunol. 2011;72(10):935-939. doi:10.1016/j.humimm.2011.06.008
17. Falleti E, Fabris C, Toniutto P, et al. Genetic polymorphisms of inflammatory cytokines and liver fibrosis progression due to recurrent hepatitis C. J Interferon Cytokine Res. 2007;27(3):239-246. doi:10.1089/jir.2006.0062
18. Gao QJ, Liu DW, Zhang SY, et al. Polymorphisms of some cytokines and chronic hepatitis B and C virus infection. World J Gastroenterol. 2009;15(44):5610-5619. doi:10.3748/wjg.15.5610
19. Gao L, Chen X, Zhang L, Wu D, Zhao H, Niu J. Association of IL-10 polymorphisms with hepatitis B virus infection and outcome in Han population. Eur J Med Res. 2016;21(1):23. Published 2016 Jun 1. doi:10.1186/s40001-016-0218-9
20. Gao QJ, Xie JX, Wang LM, Zhou Q, Zhang SY. Interaction effects among IFN-γ+874, IL-2-330, IL-10-1082, IL-10-592 and IL-4-589 polymorphisms on the clinical progression of subjects infected with hepatitis B virus and/or hepatitis C virus: a retrospective nested case-control study. BMJ Open. 2017;7(8):e013279. Published 2017 Aug 23. doi:10.1136/bmjopen-2016-013279
21. Helal SF, Gomaa HE, Thabet EH, Younan MA, Helmy NA. Impact of IL-10 (-1082) promoter-single nucleotide polymorphism on the outcome of hepatitis C virus genotype 4 infection. Clin Med Insights Gastroenterol. 2014;7:19-24. Published 2014 Apr 1. doi:10.4137/CGast.S13658
22. Jiang ZL, Zhang W, Zhang H, Liu YB, Su SB. Associations between TNF-a, TGF-B, IL-10 gene polymorphisms and hepatitis B virus infection. Chin J Infect Dis. 2010;6:592-597
23. Jiang P. 2013. Study on SNPs of IL-10 gene promoter of Qinghai Tibetan population with HBV infection. Dissertation, Qinghai University
24. Jiang F. 2017. Associations between interleukin gene polymorphisms and HBV infection. Dissertation, Chongqing Medical University
25. Karatayli SC, Ulger ZE, Ergul AA, et al. Tumour necrosis factor-alpha, interleukin-10, interferon-gamma and vitamin D receptor gene polymorphisms in patients with chronic hepatitis delta. J Viral Hepat. 2014;21(4):297-304. doi:10.1111/jvh.12139
26. Khalil H, Arfa M, El-Masrey S, El-Sherbini SM, Abd-Elaziz AA. Single nucleotide polymorphisms of interleukins associated with hepatitis C virus infection in Egypt. J Infect Dev Ctries. 2017;11(3):261-268. Published 2017 Mar 31. doi:10.3855/jidc.8127
27. Khan AJ, Saraswat VA, Choudhuri G, Parmar D, Negi TS, Mohindra S. Association of interleukin-10 polymorphisms with chronic hepatitis C virus infection in a case-control study and its effect on the response to combined pegylated interferon/ribavirin therapy. Epidemiol Infect. 2015;143(1):71-80. doi:10.1017/S0950268814000466
28. Knapp S, Hennig BJ, Frodsham AJ, et al. Interleukin-10 promoter polymorphisms and the outcome of hepatitis C virus infection. Immunogenetics. 2003;55(6):362-369. doi:10.1007/s00251-003-0594-5
29. Komatsu H, Murakami J, Inui A, Tsunoda T, Sogo T, Fujisawa T. Association between single-nucleotide polymorphisms and early spontaneous hepatitis B virus e antigen seroconversion in children. BMC Res Notes. 2014;7:789. Published 2014 Nov 6. doi:10.1186/1756-0500-7-789
30. Kusumoto K, Uto H, Hayashi K, et al. Interleukin-10 or tumor necrosis factor-alpha polymorphisms and the natural course of hepatitis C virus infection in a hyperendemic area of Japan. Cytokine. 2006;34(1-2):24-31. doi:10.1016/j.cyto.2006.03.011
31. Li YG, Liu M, Wang F, Jin L, Hong W. Genetic polymorphisms in IL-10 and IL-12b allele promoter regions in Chinese patients of Han nationality with HBV infection. World J Digestol. 2003;11(8):1139-1143
32. Li C, Zhi-Xin C, Li-Juan Z, Chen P, Xiao-Zhong W. The association between cytokine gene polymorphisms and the outcomes of chronic HBV infection. Hepatol Res. 2006;36(3):158-166. doi:10.1016/j.hepres.2006.07.007
33. Li Y. 2015. Association between host immune system genes IL-10, IL-4 and TAP polymorphisms with hepatitis C virus chronic infection. Dissertation, China Institute for Research in Biomedicine
34. Lio D, Caruso C, Di Stefano R, et al. IL-10 and TNF-alpha polymorphisms and the recovery from HCV infection. Hum Immunol. 2003;64(7):674-680. doi:10.1016/s0198-8859(03)00080-6
35. Liu J, Gao Y, Du Z, Yang B. Relationship between IL-10 gene promoter polymorphisms and outcomes of HBV infection. World J Digestol. 2010;18(16):1656-1663
36. Mangia A, Santoro R, Piattelli M, et al. IL-10 haplotypes as possible predictors of spontaneous clearance of HCV infection. Cytokine. 2004;25(3):103-109. doi:10.1016/j.cyto.2003.10.005
37. Maurya G, Hazam RK, Ruttala R, Karna R, Das BC, Kar P. A study of association between regulatory polymorphism in the IL-10 gene promoter region and acute viral hepatitis, and acute liver failure. Indian J Gastroenterol. 2018;37(4):293-298. doi:10.1007/s12664-018-0858-5
38. Minton EJ, Smillie D, Smith P, et al. Clearance of hepatitis C virus is not associated with single nucleotide polymorphisms in the IL-1, -6, or -10 genes. Hum Immunol. 2005;66(2):127-132. doi:10.1016/j.humimm.2004.11.001
39. Miyazoe S, Hamasaki K, Nakata K, et al. Influence of interleukin-10 gene promoter polymorphisms on disease progression in patients chronically infected with hepatitis B virus. Am J Gastroenterol. 2002;97(8):2086-2092. doi:10.1111/j.1572-0241.2002.05926.x
40. Moudi B, Heidari Z, Mahmoudzadeh-Sagheb H, et al. Association Between IL-10 Gene Promoter Polymorphisms (-592 A/C, -819 T/C, -1082 A/G) and Susceptibility to HBV Infection in an Iranian Population. Hepat Mon. 2016;16(2):e32427. Published 2016 Feb 20. doi:10.5812/hepatmon.32427
41. Oleksyk TK, Thio CL, Truelove AL, et al. Single nucleotide polymorphisms and haplotypes in the IL10 region associated with HCV clearance. Genes Immun. 2005;6(4):347-357. doi:10.1038/sj.gene.6364188
42. Pár A, Pár G, Tornai I, et al. IL28B and IL10R -1087 polymorphisms are protective for chronic genotype 1 HCV infection and predictors of response to interferon-based therapy in an East-Central European cohort. BMC Res Notes. 2014;7:12. Published 2014 Jan 8. doi:10.1186/1756-0500-7-12
43. Pasha HF, Radwan MI, Hagrass HA, Tantawy EA, Emara MH. Cytokines genes polymorphisms in chronic hepatitis C: impact on susceptibility to infection and response to therapy. Cytokine. 2013;61(2):478-484. doi:10.1016/j.cyto.2012.11.003
44. Peng XM, Huang YS, Ma HH, Gu L, Xie QF, Gao ZL. Interleukin-10 promoter polymorphisms are associated with the mode and sequel of HBeAg seroconversion in patients with chronic hepatitis B virus infection. Liver Int. 2006;26(3):326-333. doi:10.1111/j.1478-3231.2005.01241.x
45. Peng MW, Lu SQ, Liu J, Dong CY. Role of IL-10 polymorphisms in susceptibility to hepatitis B virus-related hepatocellular carcinoma. Genet Mol Res. 2016;15(1):10.4238/gmr.15017984. Published 2016 Mar 18. doi:10.4238/gmr.15017984
46. Pereira FA, Pinheiro da Silva NN, Rodart IF, Carmo TM, Lemaire DC, Reis MG. Association of TGF-beta1 codon 25 (G915C) polymorphism with hepatitis C virus infection. J Med Virol. 2008;80(1):58-64. doi:10.1002/jmv.21011
47. Persico M, Capasso M, Persico E, et al. Interleukin-10 - 1082 GG polymorphism influences the occurrence and the clinical characteristics of hepatitis C virus infection. J Hepatol. 2006;45(6):779-785. doi:10.1016/j.jhep.2006.07.026
48. Qiu X, Bei C, Yu H, Zeng X, Zhong Q. Study on the relationship between SNPs in IL-6, IL-10 genes and HBV-related hepatocellular carcinoma. Chin J Epidemiol. 2011;32(5):510-513
49. Ramos JA, Silva R, Hoffmann L, et al. Association of IL-10, IL-4, and IL-28B gene polymorphisms with spontaneous clearance of hepatitis C virus in a population from Rio de Janeiro. BMC Res Notes. 2012;5:508. Published 2012 Sep 17. doi:10.1186/1756-0500-5-508
50. Ren L, Lu Y, Cheng G, Yu W, Wang Z. Association between susceptibility of HBV infection and gene polymorphisms in IFN-y, TNF-a and IL-10 in the Tibetan population of Qinghai area. Prac Prev Med. 2017;24(7):814-818
51. Ribeiro CS, Visentainer JE, Moliterno RA. Association of cytokine genetic polymorphism with hepatitis B infection evolution in adult patients. Mem Inst Oswaldo Cruz. 2007;102(4):435-440. doi:10.1590/s0074-02762007005000043
52. Sepahi S, Pasdar A, Ahadi M, Gerayli S, Rostami S, Meshkat Z. Haplotype analysis of interleukin-10 gene promoter polymorphisms in chronic hepatitis C infection: a case control study. Viral Immunol. 2014;27(8):398-403. doi:10.1089/vim.2014.0024
53. Shaker OG, Sadik NA. Polymorphisms in interleukin-10 and interleukin-28B genes in Egyptian patients with chronic hepatitis C virus genotype 4 and their effect on the response to pegylated interferon/ribavirin-therapy. J Gastroenterol Hepatol. 2012;27(12):1842-1849. doi:10.1111/j.1440-1746.2012.07273.x
54. Sheneef A, Esmat MM, Mohammad AN, Mahmoud AA, Moghazy HM, Noureldin AK. Interleukin-10 and Interferon Gamma Gene Polymorphisms and Hepatitis C Virus-Related Liver Cirrhosis Risk. J Interferon Cytokine Res. 2017;37(4):175-180. doi:10.1089/jir.2016.0106
55. Fabrício-Silva GM, Poschetzky BS, de Mello Perez R, Dos Santos RC, Cavalini LT, Porto LC. Association of cytokine gene polymorphisms with hepatitis C virus infection in a population from Rio de Janeiro, Brazil. Hepat Med. 2015;7:71-79. Published 2015 Nov 2. doi:10.2147/HMER.S89447
56. Sodsai P, Surakiatchanukul T, Kupatawintu P, Tangkitvanich P, Hirankarn N. Association of cytokine and cytokine receptor gene polymorphisms with the risk of chronic hepatitis B. Asian Pac J Allergy Immunol. 2013;31(4):277-285. doi:10.12932/AP0284.31.4.2013
57. Sofian M, Kalantar E, Aghakhani A, et al. No correlation between interleukin-10 gene promoter polymorphisms and hepatitis B virus infection outcome. Hepat Mon. 2013;13(5):e8803. Published 2013 May 19. doi:10.5812/hepatmon.8803
58. Srivastava M, Ranjan A, Choudhary JK, et al. Role of proinflammatory cytokines (interferon gamma) and anti-inflammatory cytokine (interleukin-10) gene polymorphisms in chronic hepatitis B infection: an Indian scenario. J Interferon Cytokine Res. 2014;34(7):547-551. doi:10.1089/jir.2013.0054
59. Talaat RM, Dondeti MF, El-Shenawy SZ, Khamiss OA. Association between IL-10 gene promoter polymorphism and hepatitis B viral infection in an Egyptian population. Biochem Genet. 2014;52(9-10):387-402. doi:10.1007/s10528-014-9655-8
60. Tang L, Wang J, Yu R, Su J, Xu K. Associations between IL-10 gene polymorphisms and the outcomes of hepatic C virus infection among high risk populations in Jiangsu Province. Chin J Epidemiol. 2012;33(9):898-902.
61. Tang F. 2015. Association between IL-10 -819T/C, IFN-y 874T/A polymorphisms and outcomes of hepatitis B viral infection in Guangxi, China. Dissertation, Guangxi Medical University.
62. Truelove AL, Oleksyk TK, Shrestha S, et al. Evaluation of IL10, IL19 and IL20 gene polymorphisms and chronic hepatitis B infection outcome. Int J Immunogenet. 2008;35(3):255-264. doi:10.1111/j.1744-313X.2008.00770.x
63. Tseng LH, Lin MT, Shau WY, et al. Correlation of interleukin-10 gene haplotype with hepatocellular carcinoma in Taiwan. Tissue Antigens. 2006;67(2):127-133. doi:10.1111/j.1399-0039.2006.00536.x
64. Vidigal PG, Germer JJ, Zein NN. Polymorphisms in the interleukin-10, tumor necrosis factor-alpha, and transforming growth factor-beta1 genes in chronic hepatitis C patients treated with interferon and ribavirin. J Hepatol. 2002;36(2):271-277. doi:10.1016/s0168-8278(01)00243-4
65. Wang C, Shan K, He Y, Zhang T, Qi X. Associations between IL-10 -592 C/A polymorphism and hepatic B virus infection in Guizhou Province. Chin J Epidemiol. 2008;29(5):444-447.
66. Wang C, Zhang X, Zhu B, et al. Relationships between tumour necrosis factor-α, interleukin-12B and interleukin-10 gene polymorphisms and hepatitis B in Chinese Han haemodialysis patients. Nephrology (Carlton). 2012;17(2):167-174. doi:10.1111/j.1440-1797.2011.01539.x
67. Wu J, Jia Y. Association analysi of IL-10 promoter polymorphisms with hepatic B virus infection consequence. Chin J TCM on Liver Dis. 2010;20(5):262-265.
68. Xiang Y, Huang SF, Xia JR, et al. Association of the IFNAR1-17470 and IL-10-592 cytokine variants with susceptibility to chronic hepatitis B viral infections in a Chinese population. Genet Mol Res. 2014;13(4):9187-9195. Published 2014 Nov 7. doi:10.4238/2014.November.7.5
69. Xie HY, Wang WL, Yao MY, et al. Polymorphisms in cytokine genes and their association with acute rejection and recurrence of hepatitis B in Chinese liver transplant recipients. Arch Med Res. 2008;39(4):420-428. doi:10.1016/j.arcmed.2008.01.003
70. Yan Z, Tan W, Zhao W, et al. Regulatory polymorphisms in the IL-10 gene promoter and HBV-related acute liver failure in the Chinese population. J Viral Hepat. 2009;16(11):775-783. doi:10.1111/j.1365-2893.2009.01139.x
71. Yao L, Xing S, Fu X, et al. Association between interleukin-10 gene promoter polymorphisms and susceptibility to liver cirrhosis. Int J Clin Exp Pathol. 2015;8(9):11680-11684. Published 2015 Sep 1.
72. Yee LJ, Tang J, Gibson AW, Kimberly R, Van Leeuwen DJ, Kaslow RA. Interleukin 10 polymorphisms as predictors of sustained response in antiviral therapy for chronic hepatitis C infection. Hepatology. 2001;33(3):708-712. doi:10.1053/jhep.2001.22347
73. Zein NN, Germer JJ, El-Zayadi AR, Vidigal PG. Ethnic differences in polymorphisms of tumor necrosis factor-alpha, interleukin-10, and transforming growth factor-beta1 genes in patients with chronic hepatitis C virus infection. Am J Trop Med Hyg. 2004;70(4):434-437.
74. Zhang P, Li Y, Yang X. Association study on IL-10 gene promoter polymorphism related to hepatic B virus infection in Chinese Han population. Chin J Med Genet. 2006;23(4):410-414.
75. Zhu R, Gu S, Yu H. Relationships between cytokine gene polymorphism and hepatic B virus infection. Chin J Epidemiol. 2005;26(4):236-239.
76. Zhu B, Wang C, Zhang X, et al. Relationships between interleukin-12B and interleukin-10 gene polymorphisms and hepatitis C in Chinese Han hemodialysis patients. Ren Fail. 2015;37(3):505-510. doi:10.3109/0886022X.2015.1006086
